# Modulation of Neurogenesis through the Promotion of Energy Production Activity Is behind the Antidepressant-Like Effect of Colonial Green Alga, *Botryococcus braunii*

**DOI:** 10.3389/fphys.2017.00900

**Published:** 2017-11-10

**Authors:** Kazunori Sasaki, Mahmoud B. Othman, Mikihide Demura, Makoto Watanabe, Hiroko Isoda

**Affiliations:** ^1^Interdisciplinary Research Center for Catalytic Chemistry, National Institute of Advanced Industrial Science and Technology, Tsukuba, Japan; ^2^Faculty of Pure and Applied Sciences, University of Tsukuba, Tsukuba, Japan; ^3^Alliance for Research on North Africa, University of Tsukuba, Tsukuba, Japan; ^4^Algal Biomass and Energy System R&D Center, University of Tsukuba, Tsukuba, Japan; ^5^Faculty of Life and Environmental Sciences, University of Tsukuba, Tsukuba, Japan

**Keywords:** microalgae, *Botryococcus braunii*, depression, forced swimming test, dopamine synthesis, energy promotion activity, neurogenesis

## Abstract

Algae have been recognized as important resources providing functional components due to their capacity to exert beneficial effects on health. Therefore, there is increasing interest in investigating the biological activity of algae. In this study, we evaluated the antidepressant-like effect of the administration of 100 mg/kg/day of the ethanol extract of colonial green alga *Botryococcus braunii* (EEB) for 14 consecutive days in the forced swimming test (FST)-induced depression in imprinting control region (ICR) mice. Imipramine, a commercial antidepressant drug, was used as a positive control. In addition, we investigated the molecular mechanisms underlying the effect of EEB by measuring ATP production and by assessing any change in gene expression at the end of the treatment using real-time polymerase chain reaction (PCR) and microarray assays. We showed that the immobility time in the water-administered control (FST stress) group gradually increased from day 1 to day 14. However, treatment with EEB caused a significant decrease of immobility time in the FST compared with that in the FST stress group. Microarray and real-time PCR results revealed that EEB treatment induced variation in the expression of several genes associated with neurogenesis, energy metabolism, and dopamine synthesis. Interestingly, we revealed that only EEB treatment enhanced the promotion of energy production, while treatment with imipramine was ineffective. Our study provides the first evidence that *B. braunii* enhances energy production, which may contribute to the modulation of neurogenesis and to the enhancement of dopaminergic function, in turn potentially underlying the antistress- and antidepressant-like effects that we observed.

## Introduction

Stress is a part of everyday life and particularly prevalent in modern societies. There are various sources of stress in people's lives, and excessive or prolonged exposure to such stress factors has a significant impact on our wellbeing, ultimately leading to emotional and behavioral changes, together with reduced cognitive function and physical illness. Therefore, it is not surprising that stress remains a top health concern, particularly in developed countries. The damaging effect of stress on cells is well-documented; in particular, stress affects neuronal cells and may lead to the induction of neurodegenerative disorders, such as Alzheimer's disease, Parkinson's disease, and major depression. Depression is a psychiatric disorder commonly characterized by a sense of prostration; in its most severe forms, it can be life-threatening. This disorder has a worldwide prevalence of ~17% (Liu et al., 2013). Depression, which is the most common type of affective disorders, is caused by a combination of biological, psychological, social, and other factors. Depressive syndrome is characterized by significant and lasting low mood (Lu et al., 2015). In clinical practice, drug classes used for the treatment of depression include monoamine oxidase inhibitors, selective serotonin noradrenaline reuptake inhibitors (SNRIs), selective serotonin reuptake inhibitors, and tricyclic antidepressants (Bouvier et al., 2003; Fava, 2003; Shen and Liang, 2007). However, these drugs can cause a variety of adverse effects, including nausea, headaches, and nerve pain. Therefore, new and safer antidepressants, which are derived from natural products and lack side effects, need to be developed.

Algae are photosynthetic organisms that mainly inhabit the hydrosphere. They form a large and diverse group within which over 40,000 species have been described so far. However, the total number of algal species is estimated to exceed 10 million, including those that remain undiscovered and undescribed (Norton et al., 1996). Some algae and particularly microalgae have served as important sources of functional materials, such as n-3 polyunsaturated fatty acids (PUFA), vitamins, minerals, polysaccharides, and bioactive compounds (Shahidi and Janak Kamil, 2001). Moreover, microalgae are known to exhibit various biological and physiological activities, including antioxidant, anticoagulant, antiviral, and antitumoral effects (Pangestuti and Kim, 2011). Therefore, we speculated that microalgae have the potential to be a valuable natural resource of novel bioactive compounds and focused on the colonial green alga *Botryococcus braunii* Kützing (Trebouxiophyceae, Chlorophyta), which is found worldwide in freshwater and brackish lakes, reservoirs, and ponds. *B. braunii* produces large amounts of hydrocarbons, which are excreted from cells and accumulate in its colonies. Therefore, it is considered to have great potential as a renewable source of chemical products and is useful for searching for new antidepressant drugs. Moreover, to the best of our knowledge, few reports on studies exploring the physiological effects of *B. braunii* have been published.

The objectives of this study were to evaluate the antidepressant-like effect of ethanol extract of *B. braunii* (EEB), using the forced swimming test (FST) to obtain a rodent model of depression by inducing stress in imprinting control region (ICR) mice, and to understand the molecular mechanism behind the antidepressant-like effect of EEB. We also focused our attention on changes in expression levels for genes associated with neurogenesis, energy promotion activity, and dopamine synthesis. In addition, we analyzed the neuroprotective effect of EEB using rat pheochromocytoma PC12 cells.

## Materials and methods

### Preparation of ethanol extract of *B. braunii* (EEB)

A dried sample of *B. braunii* was provided by ABES, the University of Tsukuba, Japan. The dried sample was extracted using 99.5% ethanol, in the dark, and at room temperature for 2 weeks, with shaking of the mixture occurring at least once a day. At the end of the procedure, the liquid fraction (EEB) was collected, filtered through a 0.22-μm filter (Merck Millipore, Billerica, MA, USA), and used in the *in vitro* assays. For animal dosing in the *in vivo* assay, EEB was concentrated using SpeedVac (Thermo Fisher Scientific, Waltham, MA, USA) and the dried EEB was dissolved in MilliQ water.

### Animals

Male ICR mice (Charles River Laboratories Japan Inc., Yokohama, Kanagawa, Japan), 5 weeks old, weighing 35–40 g, were used for *in vivo* experiments. The animals were kept individually in cages and maintained with free access to water and food *ad libitum*, under a 12/12-h light/dark cycle. All of the experiments were carried out between 09:00 and 16:00, and the animals had been acclimatized for 7 days to the laboratory conditions ahead of the experiment. This animal experiment was approved by the Ethics Animal Care and Use Committee of the University of Tsukuba.

### Administration of ethanol extract of *B. braunii* (EEB) to ICR mice

After 1 week of acclimatization to the laboratory conditions, mice were assigned to three groups: a control group (n = 8), a group administered 20 mg/kg imipramine daily (*n* = 8), and a group administered 100 mg/kg EEB daily (n = 8). EEB dissolved in drinking water was administered orally using a tube and a syringe for 14 consecutive days. The control group was administered an equivalent volume of tap water.

Imipramine, an SNRI antidepressant drug that selectively increases dopamine and noradrenaline levels in the synaptic cleft, was used as a positive control. Imipramine was freshly dissolved in distilled water and orally administered to mice at a dose of 20 mg/kg, as reported in our previous study (Ben Othman et al., 2013).

### Forced swimming test

The FST was conducted as previously described by Ben Othman et al. (2013) To carry out the FST, we used a cylindrical jar (14 cm in diameter × 25 cm in height) filled from the bottom with 19 cm of water at 25 ± 1°C. The FST was performed at 1, 2, 6, 10, and 14 d during the period of the oral administration of EEB. Each mouse was placed gently into the water and allowed to swim freely for 5 min. The mouse was considered immobile when it showed disparity and became motionless in the water. Periods of immobility were defined as when mice only made those movements that were necessary to keep their head above the water. The duration of immobility was recorded and analyzed off-line over the last 4 min of the test.

### RNA isolation from mouse brain

Following the last FST at day 14, all mice were sacrificed by dislocation of the cervical spine and their brains were isolated. A small amount (0.1 mg) of cerebral tissue was removed and washed with ice-cold phosphate-buffered solution (PBS). The total RNA was extracted from it using the ISOGEN kit (Nippon Gene Co. Ltd., Toyama, Japan), as we reported previously (Sasaki et al., 2013). Total RNA was quantified and assessed for its quality with the NanoDrop 2000 spectrophotometer (Thermo Scientific, Wilmington, DE, USA).

### DNA microarray analysis

DNA microarray analysis was conducted on isolated RNA samples from brains treated with EEB. DNA microarray analysis was performed as reported previously (Isoda et al., 2012; Samet et al., 2015). Double-stranded cDNA was synthesized from 100 ng of total RNA with the GeneAtlas 3′ IVT Express Kit (Affymetrix Inc., Santa Clara, CA, USA). Biotin-labeled amplified RNA (aRNA) was synthesized by *in vitro* transcription using the GeneChip 3′ IVT Express Kit (Affymetrix Inc., Santa Clara, CA, USA). A total of 9.4 mg of purified aRNA was fragmented using the GeneAtlas 3′ IVT Express Kit and was hybridized for 16 h at 45°C using GeneChip MG-430 PM microarray (Affymetrix Inc., Santa Clara, CA, USA). The chip was washed and stained in the Gene Atlas Fluidics Station 400 (Affymetrix Inc., Santa Clara, CA, USA) and then the resulting image was scanned using the GeneAtlas Imaging Station (Affymetrix Inc., Santa Clara, CA, USA). Data analysis was performed using the Partek Express software (Partek Inc., St. Louis, MO, USA) provided by Affymetrix as part of their GeneAtlas system. Compared with the control (water-treated group), fold change in expression in the imipramine- or EEB-treated group was calculated and converted to log 2 data.

### Taqman quantitative RT-PCR analysis of gene expression in mouse brain

Based on the microarray analysis, reverse transcription reactions were carried out with the SuperScript III Reverse Transcriptase (RT) kit (Invitrogen, Carlsbad, CA, USA). In accordance with the manufacturer's instructions, 1 μg of total RNA and 1 μl of oligo(dT)_12–18_ primers were denatured at 65°C for 5 min and subsequently chilled at 4°C. After the addition of SuperScript III RT (200 U), the reaction mix was incubated at 42°C for 60 min, followed by another 10 min at 70°C. All primer sets and TaqMan probes for experimental genes were from Applied Biosystems (Foster City, CA, USA): mouse tyrosine hydroxylase (TH) (Mm00447557_m1), mouse pyruvate carboxylase (PC) (Mm00500992_m1), mouse brain-derived neurotrophic factor (BDNF) (Mm04230607_s1), and mouse GAPDH (Mm99999915_g1). For the quantification of mRNA, TaqMan Real Time-PCR amplification reactions were carried out using an AB 7500 Fast Real-Time PCR system (Applied Biosystems). Amplifications were performed in a final volume of 20 μl, using 10 μl of TaqMan Universal PCR Master Mix UNG (Applied Biosystems), 1 μl of the corresponding primer/probe mix, and 9 μl of template cDNA (final concentration 100 ng/20 μl). Cycling conditions were as follows: 2 min at 50°C, 10 min at 95°C, and 40 cycles at 95°C for 15 s followed by 60°C for 1 min.

### Measurement of brain ATP content

ATP levels in the bupropion tissues were measured with firefly bioluminescence using a luminescence luciferase assay kit (TOYO Ink, Tokyo, Japan). A small amount (0.1 mg) of cerebral tissue was homogenized with 10 mL of ice-cold homogenate buffer (0.25 M sucrose, 10 mM HEPES-NaOH, pH 7.4). After centrifugation, the supernatant was collected and 100 μL of it was transferred to a 96-well plate. After adding 100 μL of luciferin-luciferase solution (TOYO Ink, Tokyo, Japan), 150 μL of the mixed solution was transferred to another 96-well plate and incubated for 10 min. After 10 min of incubation, light emission was recorded using a luminometer (Powerscan HT; Dainippon Pharmaceutical, Osaka, Japan).

### Cell culture

PC12 cell culture and MTT assay were performed in accordance with our previous work (Sasaki et al., 2013). PC12 cells (RIKEN, Tsukuba, Japan) were cultured in 75 cm^2^ flasks (BD Biosciences, San Jose, CA, USA) and maintained in Dulbecco's Modified Eagle's Medium (DMEM) (Sigma-Aldrich, St. Louis, MO, USA) containing 10% heat-inactivated horse serum (Gibco, Yokohama, Japan), 5% fetal bovine serum (Sigma-Aldrich), and supplemented with 100 U·ml^−1^ penicillin and 100 μg·ml^−1^ streptomycin (ICN Biomedicals, Tokyo, Japan), in a water-saturated 5% CO_2_ atmosphere at 37°C. For the experiments in this study, cells were used between passage 3 and passage 8.

### MTT assay for neuroprotection

Cell viability was measured using the 3-(4,5-dimethylthiazol-2-yl)-2,5-diphenyltetrazolium bromide (MTT) assay. PC12 cells (1 × 10^5^ cells·ml^−1^) cultured in a 96-well plate (BD Biosciences) were pretreated with 1/1,000 dilution of EEB for 10 min, followed by the addition of 200 μM corticosterone (Sigma-Aldrich, St. Louis, MO, USA) for 48 h. After sample treatment, 100 μl of culture medium and 10 μl of MTT (5 mg·ml^−1^) were added and the cells were incubated for 6 h. The MTT formazan formed was dissolved in 100 μl of 10% SDS (w/v) and the absorbance was measured using a microtiter plate reader (Powerscan HT; Dainippon Sumitomo Pharma Co. Ltd., Osaka, Japan).

### Statistical analysis

Results are expressed as mean ± standard error of the mean (SEM). Statistical analysis of the results obtained in the FST was carried out using two-way ANOVA with Ryan-einot-gabriel-welsch multiple range test. One-way ANOVA followed by Ryan-einot-gabriel-welsch multiple range test was also used. The statistical evaluation was performed using the Student's *t*-test between control and corticosterone-treatment group in *in vitro* experiment. A *P* < 0.05 was considered statistically significant.

## Results

### EEB reversal of depression-like behavior induced by FST

**Figure 1:**
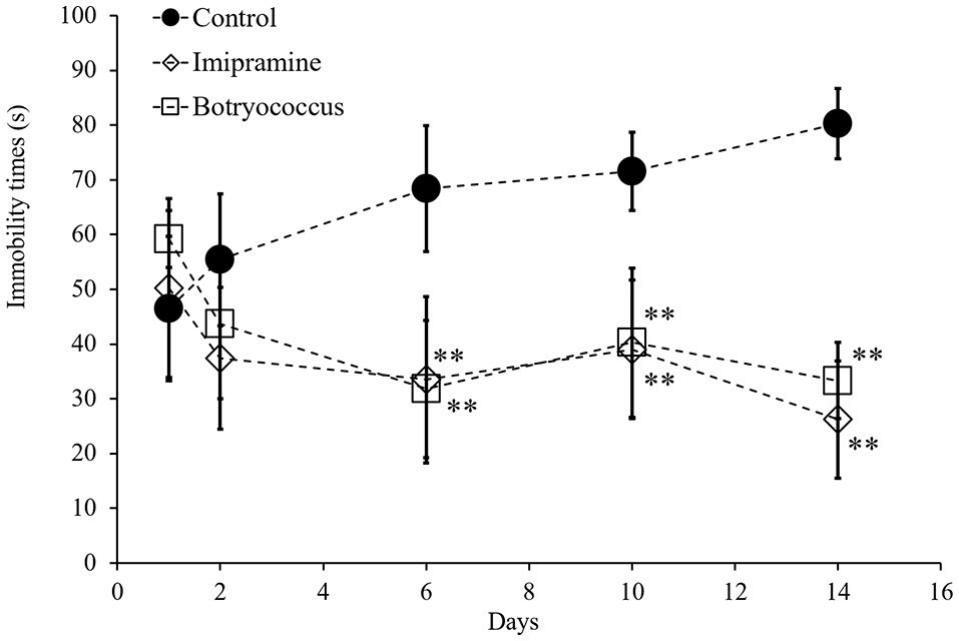
Effects of administration of ethanol extract of *Botryococcus braunii* (EEB) on the immobility time in the forced swimming test (FST). Mice were orally administered water (control), imipramine (20 mg/kg), and EEB (100 mg/kg) daily for 14 consecutive days. FST was carried out on days 1, 2, 6, 10, and 14. The immobility time during the final 4 min of a 5-min total session was measured. FST immobility time measured for each group. Each data point expressed as the means ± SEM (*n* = 8) and were analyzed by two-way ANOVA followed by Ryan-einot-gabriel-welsch multiple range test, ^**^P < 0.01 vs. control group.

FST is a behavioral animal model that has been widely adopted for investigating depression. To determine whether EEB has antidepressant-like activity, its effect on FST-induced stress in mice was investigated. The administration of 100 mg/kg EEB for 14 consecutive days caused no mortality or significant body weight change in any animal. As shown in Figure 1, the immobility time in the water-administered control (FST stress) group gradually increased from day 1 to day 14 (*D* = day; D1, 46.5 ± 13.2 s; D2, 55.4 ± 12.0 s; D6, 68.4 ± 11.5 s; D10, 71.5 ± 7.14 s; D14, 80.3 ± 6.40 s; *P* < 0.05). However, this trend was not observed in the imipramine- and EEB-administered groups.

At day 14, the average immobility time for the EEB-administered group (33.3 ± 7.0 s) was similar to that in the imipramine-administered group (26.3 ± 10.7 s), which represented our positive control (Figure 1). In mice treated with imipramine, EEB induced a three-fold reduction of the average immobility time compared with that in the water-administered control mice (80.3 ± 6.40 s; *P* < 0.01).

### EEB-induced variation of energy metabolism-, dopamine production-, and neurogenesis-related gene expression

To evaluate the molecular mechanism behind the antidepressant-like effect of EEB, we performed microarray analysis of ICR mouse cerebrum to investigate changes in gene expression. We found that the expression of eight genes was altered in the ICR mice administered EEB compared with their levels in the control group (Table 1). These included the short stature homeobox 2 (Shox2), paired-like homeodomain transcription factor 2 (Pitx2), teashirt zinc finger family member 1 (Tshz1), and LIM homeobox protein 9 (Lhx9) genes associated with neurogenesis (*P* < 0.05; compared with the control group). Moreover, the expression of the polyribonucleotide nucleotidyltransferase 1 (Pnpt1) gene associated with energy metabolism was upregulated, while that of the arrestin domain containing 4 (Arrdc4) gene involved in the regulation of ATP production was downregulated (*P* < 0.05; compared with the control group). In addition, the expression of two genes associated with dopamine synthesis was modulated: the arginine/serine-rich coiled-coil 1 (Rsrc1) gene was upregulated, while the protein phosphatase 1, regulatory (inhibitor) subunit 1B (Ppp1r1b) gene was downregulated (*P* < 0.05; compared with the control group). A similar pattern of alterations of expression for the genes involved in neurogenesis and dopamine synthesis was observed in the imipramine-administered group. In summary, our results show that the administration of EEB induced variation in the expression of energy metabolism-, dopamine production-, and neurogenesis-related genes.

**Table 1:** Classification of the modulated genes and their fold change in expression in imipramine- and EEB-administered imprinting control region mice in comparison to the control, as identified by DNA microarray analysis.

**Gene symbol**	**Gene name**	**Fold change (Control vs. imipramine)**	**Fold change (Control vs. EEB)**	**Molecular function**	**Associated phenotype**
Shox2	Short stature homeobox 2	2.41^**^	3.09^**^	Plays an important role during the cerebellar neurogenesis, by maintaining the bone morphogenetic protein expression levels (Rosin et al., 2015)	Neurogenesis
Pitx2	Paired-like homeodomain transcription factor 2	1.84^*^	2.37^*^	Necessary for the normal development of the subthalamic nucleus	
Tshz1	Teashirt zinc finger family member 1	2.24^**^	2.02^**^	Maintain the expression of the prokineticin receptor 2, a G protein–coupled receptor essential for OB development and related to prokineticin signaling (Ragancokova et al., 2014)	
Lhx9	LIM homeobox protein 9	1.69^**^	1.39^*^	The typical marker genes of neurogenesis at the stage of immature neuronal cells (Ng et al., 2005).	
Pnpt1	Polyribonucleotide nucleotidyltransferase 1	1.06	2.05^**^	Gene encoding for PNPase, associate with Mitochondrial importation of nucleus-encoded RNAs (Kamenski et al., 2007; Tarassov et al., 2007; Duchene et al., 2009; Smirnov et al., 2011).	Energy Promotion
Arrdc4	Arrestin domain containing 4	−1.08	−1.33^**^	Negative regulator of glucose uptake (Patwari et al., 2009)	
Rsrc1	Arginine/serine-rich coiled-coil 1	1.63^**^	1.49^**^	Function in dopamine, glutamate, and fibroblast growth factor receptor signaling (Potkin et al., 2009)	Dopamine synthesis
Ppp1r1b	Protein phosphatase 1, regulatory (inhibitor) subunit 1B	−3.83^**^	−5.54^**^	Involved in the regulation of dopaminergic and glutaminergic signaling (Albert et al., 2002; Svenningsson et al., 2002)	

*Table values are expressed as mean ± SEM for three mice in each group*.

* *P < 0.05*,

** *P < 0.01 in comparison to control mice*.

### EEB-induced upregulation of BDNF, TH, and PC gene expression in ICR mouse cerebrum

**Figure 2:**
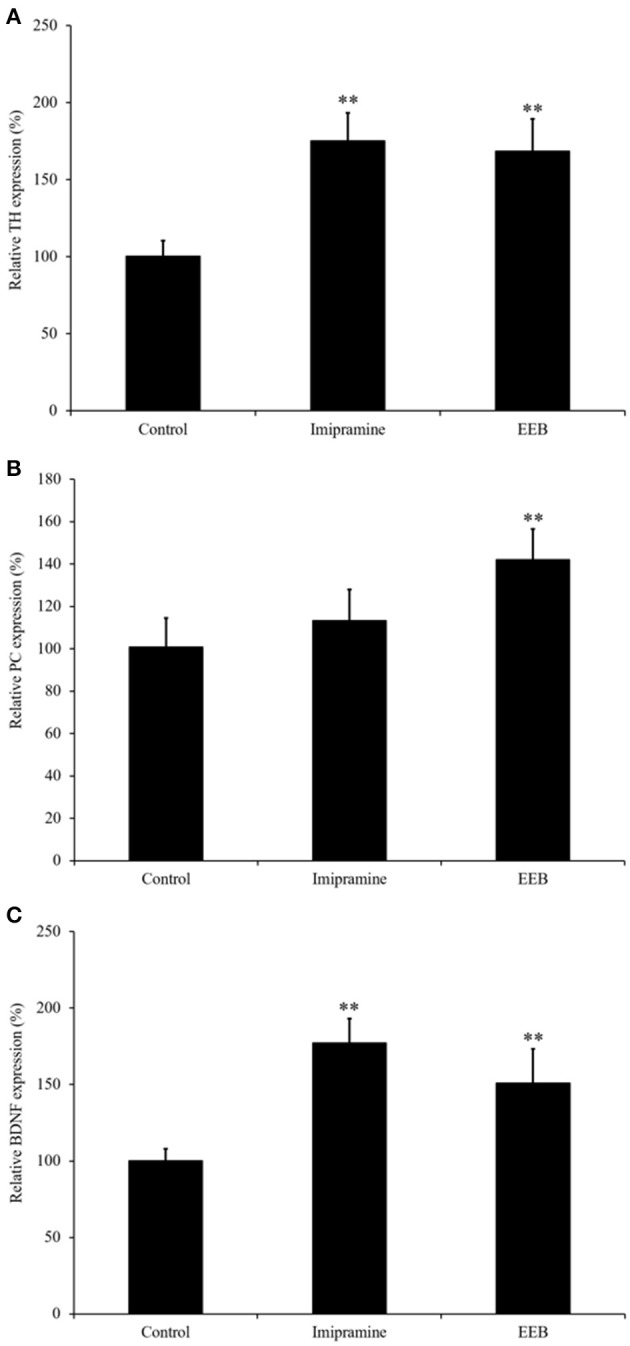
Effect of the administration of ethanol extract of *Botryococcus braunii* (EEB) on mRNA expression of pyruvate carboxylase (PC), brain-derived neurotrophic factor (BDNF), and tyrosine hydroxylase (TH) in imprinting control region mouse cerebrum. Mice were orally administered water (control), imipramine (20 mg/kg), and EEB (100 mg/kg) daily for 14 consecutive days. Gene expression levels of TH **(A)**, PC **(B)**, and BDNF **(C)** were normalized to the GAPDH level and were expressed as the ratio of that in the control group. Values are expressed as the means ± SEM (*n* = 3 independent experiments) and were analyzed by one-way ANOVA followed by Ry8an-einot-gabriel-welsch multiple range test, ^**^P < 0.01 vs. control group.

Based on the results obtained from the microarray analysis, we investigated the mRNA expression levels of TH, PC, and BDNF in the ICR mouse cerebrum from the four experimental groups. Real-time (RT) PCR results (Figure 2) showed that the mRNA expression levels of TH were significantly upregulated in the EEB-administered groups (168.6 ± 21.0%, compared with the control group; *P* < 0.01; Figure 2A). The mRNA expression levels of PC and BDNF were also upregulated in the EEB-administered groups (PC: 142 ± 14.6% compared with the control group, *P* < 0.01; BDNF: 151.1 ± 22.4% compared with the control group, *P* < 0.01; Figures 2B,C). The administration of imipramine induced the overexpression of TH and BDNF, while it did not affect the mRNA level of PC (Figure 2).

### EEB-induced production of ATP in ICR mouse cerebrum

**Figure 3:**
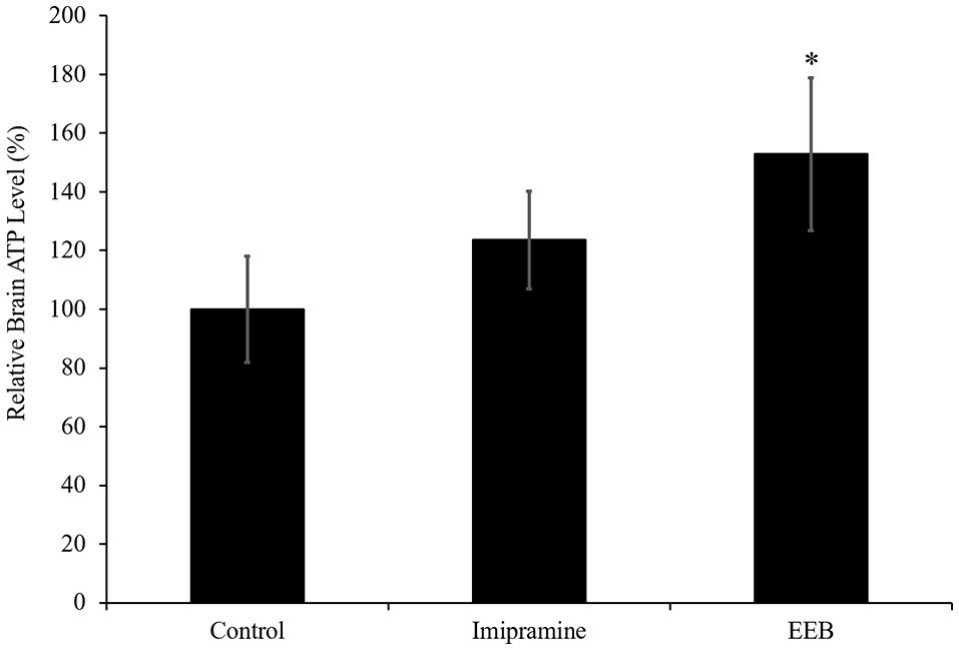
Effects of the administration of ethanol extract of *B. braunii* (EEB) on the ATP levels in imprinting control region mouse cerebrum. Mice were orally administered water (control), imipramine (20 mg/kg), and EEB (100 mg/kg) daily for 14 consecutive days. Values are expressed as the means ± SEM (*n* = 4 independent experiments) and were analyzed by one-way ANOVA followed by Ryan-einot-gabriel-welsch multiple range test, ^*^P < 0.05 vs. control group.

The results obtained from the microarray analysis and RT-PCR showed that the upregulation of PC expression occurred in the EEB-administered groups, but not in the imipramine-administered mice. This indicates that EEB may increase the energy level of mice. Therefore, we determined the ATP content in the brains of the mice administered EEB. ATP is a multifunctional nucleotide referred to as the “molecular unit of currency” of intracellular energy transfer. It is also widely used as a marker of cell proliferation as it transports the chemical energy necessary for metabolic activity into the cells. The levels of ATP production in the EEB-administered mice were measured using a luciferase method. Mice administered EEB had significantly upregulated levels of luminescence (152.8 ± 25.9%) compared with the control group (*P* < 0.05; Figure 3). In contrast, ATP levels of imipramine-administered mice were not significantly altered (Figure 3).

### EEB-mediated protection against corticosterone-induced cell death

**Figure 4:**
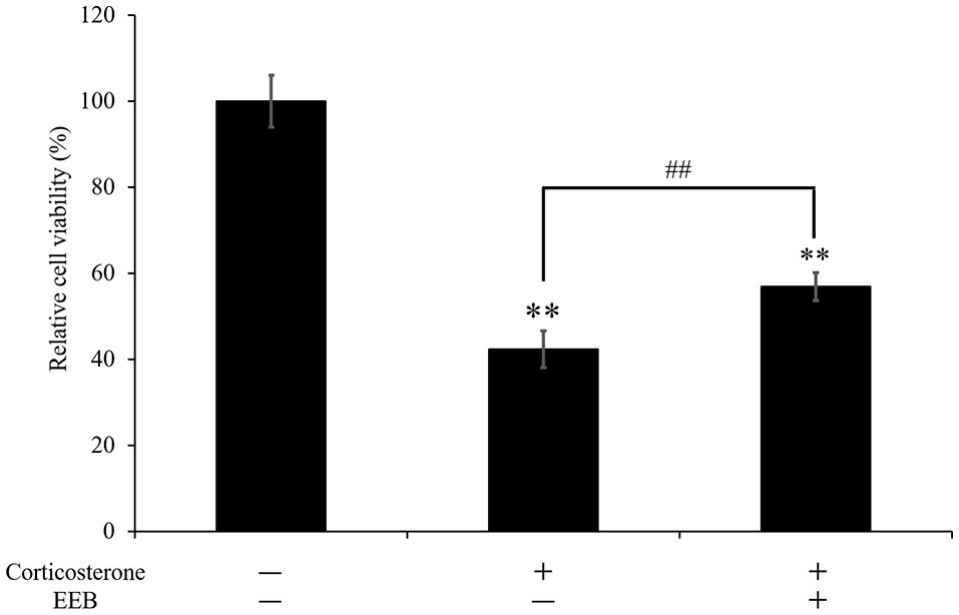
Effect of ethanol extract of *B. braunii* (EEB) on the corticosterone-induced changes in PC12 cell viability. Values are expressed as the means ± SEM (*n* = 5 independent experiments) and were analyzed by one-way ANOVA followed by Ryan-einot-gabriel-welsch multiple range test, ^**^P < 0.01 vs. control cells, ^##^*P* < 0.01 vs. corticosterone-treated cells.

We next performed the MTT assay to investigate the viability of PC12 cells after treatment with corticosterone. Application of 200 μM corticosterone significantly decreased cell viability to 42.4 ± 4.3% compared with that of untreated cells (*P* < 0.01; Figure 4). However, pretreatment with a 1/1,000 dilution of EEB for 10 min reversed this corticosterone-induced cell death, resulting in a significant increase of cell viability by 34.3 ± 3.2% compared with that in the corticosterone-treated group” and revise (*P* < 0.01; Figure 4).

## Discussion

The present study was conducted to investigate the potential antidepressant-like effect of the microalga *B. braunii* and to elucidate the mechanism behind this effect. It is estimated that about 72,500 algal species have been characterized globally (Guiry, 2012). Algae produce bioactive secondary metabolites that include polyphenolic compounds, polysaccharides, steroids, fatty acids, carotenoids, mycosporine-like amino acids, halogenated compounds, polyketides, lectins, peptides, and their derivatives (Faulkner, 2001; Cardozo et al., 2007). Among the large number of biological functionalities of marine algal natural products, antioxidant, anti-inflammatory, anticancer, immunomodulatory, antidiabetic, antimicrobial, anticoagulant, tyrosinase inhibitory, and UV-protective effects have been highlighted, based on extensive studies (Blunt et al., 2015). However, there have been no reports on the antidepressant-like effect of *B. braunii*. Therefore, the discovery of novel antidepressants from these microalgae could provide new insight in the fields of biomedical and pharmaceutical research.

The FST is a standard animal model for behavioral measurement of depression. In this test, the degree to which the animal ceases to struggle and becomes relatively passively immobile is used to assess the depressive behavior. Immobility displayed in this behavioral model has been hypothesized to reflect behavioral despair, which may mimic depressive disorders in humans. Moreover, there is a significant correlation between the clinical potency of antidepressants and the potency of the same drugs in this model. Therefore, FST is usually adopted to screen or evaluate antidepressant drugs (Porsolt et al., 1978). The current study showed that the duration of immobility in the FST was significantly decreased after the administration of 100 mg/kg EEB, indicating that *B. braunii* has antidepressant effects. Furthermore, the oral administration of *B. braunii* showed a comparable effect to that of imipramine, a commercial antidepressant drug.

Microarray studies have been utilized as a useful tool to characterize depression-related biomarkers in patients as well as to further our understanding of the mechanisms underlying depression in rodent models of this disease. In this study, we identified eight genes that may be related to the pathophysiology of depressive-like behavior induced by FST-related stress. Three biological pathways (I: dopamine synthesis; II: energy metabolism; III: neurogenesis) were identified to be significantly altered in EEB-administered groups compared with their levels in the control group.

As proposed by Li et al. (2015), the monoamine hypothesis suggests that major depression might result from the dysregulation of monoaminergic neurotransmitters such as dopamine, serotonin, and norepinephrine in the central nervous system. Dopamine is the most abundant monoamine neurotransmitter in the brain, and plays a critical role in the regulation of emotions, motivation, cognition, reward circuits, and reinforcement behavior (Nieoullon and Coquerel, 2003; Wise, 2004; Perkins et al., 2008). In this study, microarray results showed that EEB induced downregulation of the Ppp1r1b gene, which encodes the dopamine- and cAMP-regulated neuronal phosphoprotein (DARPP-32). DARPP-32 is involved in the regulation of dopaminergic and glutaminergic signaling, and it has been implicated in various neurological and psychiatric disorders including schizophrenia (Albert et al., 2002) and depression (Svenningsson et al., 2002). In addition, EEB also induced overexpression of the Rsc1 gene, which functions in dopamine, glutamate, and fibroblast growth factor receptor signaling (Potkin et al., 2009). Moreover, we determined the mRNA expression of tyrosine hydroxylase (or tyrosine 3-monooxygenase, TH), the rate-limiting enzyme in the biosynthesis of the catecholamine neurotransmitters dopamine (DA) and norepinephrine (NE), and of adrenaline in neurons. The regulated activity of TH is thought to play a critical role in modulating the functional activity of the dopaminergic neuronal systems in the brain (Fu et al., 2006). Catecholamines, such as DA and NE, are known to play an important role in several behavioral and neurodegenerative diseases such as depression and Parkinson's disease. In our study, we demonstrated that EEB treatments increased the level of TH by 168.6 ± 21.0%, in comparison to that in vehicle-treated animals. Therefore, our results demonstrate that *B. braunii* may act as an antidepressant by enhancing catecholamine synthesis in the brain.

Energy metabolic pathways [including glycolysis/gluconeogenesis, the tricarboxylic acid (TCA) cycle, and oxidative phosphorylation] have been studied extensively in major depression disorder (Reininghaus et al., 2013). In our study, we reported the variation in the expression of Pnpt1 and Arrdc4 genes, which are involved in energy production. The Pnpt1 gene product is a polynucleotide phosphorylase (PNPase), an enzyme associated with the import of nucleus-encoded RNAs such as transfer RNA (tRNA), 5S ribosomal RNA (5S rRNA), ribonuclease P RNA (RNase P RNA), and MRP (mitochondrial RNA processing) RNAse into mitochondria. These nucleus-encoded RNAs are essential for mitochondrial DNA replication, transcription, and translation (Kamenski et al., 2007; Tarassov et al., 2007; Duchene et al., 2009; Smirnov et al., 2011). It is reported that deficiency of PNPase leads to cellular changes secondary to mitochondrial dysfunction, which include lactate accumulation, reduction in steady-state ATP levels, and reduced cell proliferation (Chen et al., 2006). The expression of Arrdc4, a negative regulator of glucose uptake (Patwari et al., 2009), was also downregulated in the cerebrum of EEB-administered ICR mice. Glucose is a fundamental nutrient, providing ATP for energy as well as carbon for biosynthesis (Vander Heiden et al., 2009). In addition, from the microarray results, we determined the gene expression of pyruvate carboxylase (PC), the rate-limiting enzyme catalyzing the ATP-dependent carboxylation of pyruvate to oxaloacetate. Our findings demonstrate that EEB may have an antidepressant-like effect by enhancing the level of energy availability. Interestingly, we demonstrated that only EEB treatments increased PC expression and ATP level in mouse cerebrum, while treatment with imipramine was ineffective on both of these parameters.

It is reported that ATP can induce an increase of BDNF expression (Klein et al., 2012). In our study, we demonstrated that EEB significantly increased BDNF gene expression in mouse brain. BDNF is essential for neurogenesis and for the rearrangement of axonal arbors in the brain (Jeanneteau et al., 2010). Furthermore, it has been shown that the exogenous application of BDNF to hippocampal neurons alleviates the neurotoxic insult induced by corticosterone (Nitta et al., 1999). In our *in vitro* experiment, we confirmed that 200 μM corticosterone induced a significant decrease in the viability of PC12 cells. However, EEB attenuated the cell death induced by corticosterone. This result indicates a close functional relationship between BDNF and glucocorticoids, and may explain the important role of BDNF in the modulation of major depressive disorder. In addition to our findings, previous publications demonstrated that the promotion of neurogenesis in mouse hippocampus could be the mechanism underlying the antidepressant-like effect of some drugs (Ito et al., 2008). Moreover, it was shown that compounds with an antidepressant-like effect in animal models of chronic unpredicted stress restored the levels of BDNF expression in hippocampal neurons and astrocytes (Jin et al., 2013).

Our results from the microarray analysis also revealed that EEB treatment induced the overexpression of a few genes related to the process of neurogenesis (Shox2, Pitx2, Tshz1, and Lhx9). Shox2 plays an important role during cerebellar neurogenesis, by maintaining the proper balance of morphogen sonic hedgehog and bone morphogenetic protein (BMP) expression levels (Rosin et al., 2015). The Pitx2 gene product is necessary for the normal development of the subthalamic nucleus and its projections to the tegmentum, as well as for the development of the projections arising in the midbrain. Pitx2 protein is expressed in postmitotic neurons of the midgestation central nervous system, including GABAergic neurons of the future thalamus and midbrain, and neurons of the subthalamic nucleus (Martin et al., 2002). Moreover, Pitx2 is required to establish functional connectivity of these terminally differentiated neurons (Martin et al., 2004). Tshz1 is expressed in the developing olfactory bulb (OB) and the dorsolateral ganglionic eminence (Caubit et al., 2005). It is reported that the major function of Tshz1 in the OB is to maintain the expression of prokineticin receptor 2, a G protein-coupled receptor essential for OB development and related to prokineticin signaling (Ragancokova et al., 2014). Prokineticin signaling is involved in several biological processes, including nociception, circadian rhythm, and neurogenesis (Ng et al., 2005; Hu et al., 2006; Li et al., 2012). Lhx9 was isolated by degenerate RT-PCR followed by mouse embryonic library screening. Lhx9 is one of the typical marker genes of neurogenesis at the stage of immature neuronal cells (Peukert et al., 2011). These results suggest that EEB treatment induced the enhancement of neurogenesis through the promotion of energy production.

The results from our study showed that *B. braunii* has an antidepressant-like effect in ICR mice with FST-induced depression. This microalga may contain proteins, carbohydrates, lipids, or bioactive molecules such as n-3 PUFA and carotenoids that exert an antidepressant-like effect. And, *B. braunii* produces carotenoids including β-carotene, lutein, violaxanthin, canthaxanthin, astaxanthin, and zeaxanthin (Ranga Rao et al., 2006, 2010). A number of reports have been published on studies in which attempts were made to elucidate the antidepressant effect of these active substances. For example, astaxanthin was shown to improve the impaired behavior in an animal model of autism, presumably by its antioxidant activity (Al-Amin et al., 2015). Additionally, n-3 PUFAs are under investigation for their possible use in the treatment and prevention of depression. An early study in rodents demonstrated that a reduction of dietary n-3 PUFA intake induced a decrease in the levels of nerve growth factor (NGF) in the hippocampus, and that NGF levels in different brain regions were affected differently by dietary n-3 PUFA deficiency and restoration (Ikemoto et al., 2000). Interestingly, meta-analysis of clinical trials using a combination of different types of n-3 PUFA, such as EPA and DHA, supplementation in patients with affective disorders and depression showed the therapeutic benefit of this combination for the amelioration of symptoms in patients affected by major depressive disorder (Grosso et al., 2014). Therefore, *B. braunii*, which is rich in n-3 PUFA and carotenoids, may be useful as a new therapeutic agent for depression. Further studies on the chemical composition of *B. braunii* will be necessary.

## Conclusion

In summary, we report here for the first time evidence showing that *B. braunii* exerts an antidepressant-like effect in an animal model of depression. This appears to be mediated by the enhancement of energy promotion, neurogenesis, and dopamine synthesis in the brain. The observed effects of this alga showed in terms of significant differences compared with imipramine, a commercial antidepressant drug that was used as a positive control in this study, indicating a difference in the molecular mechanism underlying their antidepressant effects. These molecular mechanisms are currently not understood and will be the focus of our future research.

## Author contributions

KS, MO, and HI conceived and designed the experiments; KS and MO performed the experiments; KS prepared the figures and tables; KS analyzed and interpreted the results, and wrote the paper; MD and MW provided *B. braunii*; and HI edited and revised the manuscript.

### Conflict of interest statement

The authors declare that the research was conducted in the absence of any commercial or financial relationships that could be construed as a potential conflict of interest.
